# Diluted Acetic Acid Softened Intermuscular Bones from Silver Carp (*Hypophthalmichthys molitrix*) by Dissolving Hydroxyapatite and Collagen

**DOI:** 10.3390/foods11010001

**Published:** 2021-12-21

**Authors:** Yueyue Liu, Huiman Jiang, Longteng Zhang, Yuqing Tan, Yongkang Luo, Hui Hong

**Affiliations:** 1 Beijing Laboratory for Food Quality and Safety, College of Food Science and Nutritional Engineering, China Agricultural University, Beijing 100083, China; liuyueyue_cau@163.com (Y.L.); 18989197032@163.com (H.J.); zhanglongteng92@163.com (L.Z.); yuqingtan@cau.edu.cn (Y.T.); luoyongkang@cau.edu.cn (Y.L.); 2 Xinghua Industrial Research Centre for Food Science and Human Health, China Agricultural University, Xinghua 225700, China

**Keywords:** silver carp, intermuscular bones, acetic acid, softening mechanism

## Abstract

Intermuscular bones (IBs) pose physical hazards that threaten consumer health and food safety. This study aimed to investigate the mechanism of softening IBs from silver carp with diluted acetic acid. IBs (separated from muscle) and fillets (without removing IBs) were treated with diluted acetic acid. Analyses of sensory attributes and the hardness of treated IBs indicated that diluted acetic acid (<10 mmol/L) could soften IBs effectively. Additionally, 0.5 mmol/L acetic acid softened IBs within fillets without significantly affecting the texture and flavor of fillets. Analyses of microstructure, minerals (calcium and phosphorus) and collagen content, and the Fourier transform infrared (FTIR) spectra of IBs indicated that acetic acid broke connections (formed by collagen that shared hydroxyl groups) between collagen molecules, and between collagen and hydroxyapatite (HAP), thus inducing the dissolution of collagen and HAP. The dissolution of HAP contributed more to IBs softening than collagen.

## 1. Introduction

Silver carp (*Hypophthalmichthys molitrix*) is a species of freshwater fish that is native to China and Eastern Siberia. The worldwide production of silver carp was 4.83 million tonnes in 2019, which ranked number two after the production of grass carp (*Ctenopharyngodon idella*) (5.7 million tonnes) [1]. Silver carp is an economically important fish species in China due to its high nutritional value and low price. However, silver carp is a major invasive species in the U.S., and it is rarely consumed by Americans due to its numerous intermuscular bones (IBs) and off-flavors. IBs are among the important physical hazards that threaten consumer health and food safety [2] due to their potential to cause gastrointestinal mucosal damage, intestinal perforation, thyroid abscesses, and even death if they are accidentally ingested [3]. In addition, IBs negatively affect dining experiences and are unlikely to be removed through filleting. Therefore, the elimination of the hazards of IBs from fish fillets is important for improving meat quality and its commercial value.

The main components of IBs are collagen, minerals that contain hydroxyapatite (Ca_10_(PO_4_)_3_(OH)_2_, HAP), and water [4]. Collagen has a triple-helix structure with three polypeptide α-chains. More than 27 forms of collagen are distributed in animal tissues, and type I collagen is the most abundant type of collagen in bones, accounting for approximately 95% of the entire collagen content in bones [5]. HAP is the main form of calcium and phosphorus present in bones [6]. In the structure of bones, nuclei of HAP crystal platelets are located in the hole-zones of collagen. The nucleus grows in length along the collagen’s long axis and in width along the channels [7]. HAP crystals keep growing until they emerge from the holes and completely cover the collagens; therefore, HAP is present both inside and outside the collagen [8,9]. Collagen affects the toughness of bones, but bone stiffness and strength are predominantly dependent on mineral content [10]. It is postulated that IBs could be softened by reducing the content of HAP and collagen.

Acid extraction (usually acetic acid) is an effective way to extract collagen from bones at low temperatures. Caputo et al. [11] proposed an acetic acid-based method to extract collagen from bones, reporting that the yield from a 0.5 mol/L acetic acid extraction was much higher than the yield extracted using 0.6 mol/L HCl. Kittiphattanabawon et al. [12] reported an effective collagen extraction strategy using 0.5 mol/L acetic acid from bones and the skin of a bigeye snapper (*Priacanthus tayenus*). However, the diluted acetic acid (less than 0.5 mol/L) used for collagen extraction from bones was rarely reported. In general, heat treatment is a conventional method used to extract HAP from bones. Venkatesan and Kim [13] evaluated the effect of temperature, ranging from 200 to 1200 °C, on the isolation and characterization of HAP from tuna (*Thunnus obesus*) bones. They found that the optimal temperatures to obtain HAP with optimal physicochemical properties (i.e., HAP that is highly pure and suitable for further applications) was between 600–900 °C. In addition to this heat treatment, the acid-based extraction of HAP is also an alternative method. Liu and coworkers [14] proposed a method for extracting HAP from bighead carp (*Aristichthys nobilis*) scales by using a solvent that consisted of choline chloride and acetic acid (at a ratio of 1:2 (*v*/*v*)). Shimaosaka, Shimomura, and Minoru [15] reported that minerals in horse mackerel (*Trachurus japonicus*) bones exhibited a rapid elution when the bones were cured in 4% acetic acid. Acetic acid is also used to preserve meat quality. For example, acetic acid decreased the pH of vacuum-packaged meat and slowed down the growth of *Carnobacterium* spp., which prolonged shelf life [16]. Washing with 2% acetic acid prevented the growth of pathogens on turkey roll (*Listeria monocytogenes*) and beef plate (*Escherichia coli* O157:H7) [17]. McDermott et al. [18] reported that immersion in 5% acetic acid extended the shelf life of white crab (*Cancer pagurus*) meat to 8–11.5 days. Therefore, the extraction of collagen and HAP from separated IBs and whole fillets (without removing IBs) through acetic acid treatment is a promising strategy to soften IBs because acetic acid is edible and might be beneficial for the preservation of fillets.

Previous studies mainly focused on the extraction of collagen and/or HAP from frame bones using acetic acid, and the softening mechanism of IBs by immersion in acetic acid remains unclear. The objective of this study was to provide a deeper insight into the acetic acid-based softening of IBs. All IBs from silver carp were collected from dorsal muscles and their morphology was observed. The hardness and microstructure of IBs after immersion in acetic acid (in different concentrations) were also determined. We also evaluated the sensory attributes of acetic-acid-treated fillets (flavor and texture) and acetic acid-softened IBs both in vitro and in vivo (i.e., sharpness and hardness). In addition, the calcium, phosphorus, and collagen content and Fourier transform infrared (FTIR) spectra of IBs were measured.

## 2. Materials and Methods

### 2.1. Raw Materials and Preparation of IBs

Twelve silver carp with average weight and length of 1.52 ± 0.21 kg and 50.32 ± 2.22 cm, respectively, were purchased from a local aquatic market (Beijing, China) and transported to the laboratory in water with oxygen. After they arrived, fish were stunned by physical blows to the head. Then, silver carp were scaled, gutted, decapitated, washed, and then filleted. Fillets were washed in running tap water and placed onto a stainless-steel frame for draining. The entire experimental procedure was in accordance with the Guidance on Treating Experimental Animals developed by China’s Ministry of Science and Technology in 2006 and regulations issued by China State Council in 1988 [19]. After draining, fillets were divided into two parts: (i) three of them were cut into 5 mm thick pieces, vacuum-packaged in polyethylene bags, and stored at 4 °C prior to further acetic acid immersion, in order to investigate the softening effect of acetic acid on in vivo IBs; (ii) twenty-one fillets were put into polyethylene bags and heated in a water bath (97 °C) for 10 min. After cooling cooked fillets at room temperature, all IBs were isolated and mixed, and the morphology of IBs were observed. The weight of IBs used in each group were 20 mg, they were picked from the mixed IBs randomly. The isolated IBs were immersed in 0, 0.05, 0.1, 0.25, 0.5, 1, 5, and 10 mmol/L acetic acid solutions at 4 °C for 12 h. The ratio of IBs to the solution was 1:500 (*w*/*v*). Softened IBs were stored at −20 °C until further analysis. Fillets (100 g) from part (i) were immersed in acetic acid (0, 0.25, 0.5, 0.75, and 1 mmol/L) at 1:20 (*w*/*v*) for 12 h at 4 °C. The concentrations of acetic acid used for fillets were selected according to the acetic acid softening effect on IBs. Fillets were then immersed in deionized water for 12 h at 4 °C to rinse off the residual acid.

### 2.2. Chemical Reagents

All reagents used were of analytical grade unless otherwise specified, Food grade vinegar was used to treat IBs for sensory evaluation. A hydroxyproline assay kit (No. A030-3-1) was purchased from Nanjing Jiancheng Bioengineering Institute (Nanjing, China).

### 2.3. Number and Morphology of IBs

All IBs isolated from a whole dorsal muscle of carp were arranged in order and photographed.

### 2.4. Microstructure

The microstructures of IBs treated with acetic acid solutions (or deionized water) were observed using scanning electron microscopy (SEM) (SUPR55, ZEISS, Jena, Germany). The sample preparation was based on the method of Fiedler et al. [20] and Rubin et al. [9] with some modifications. The cut IBs (2 mm) were fixed for 24 h in 2.5% (*v*/*v*) glutaraldehyde solution. After fixation, IBs were dehydrated with a gradient of ethanol (50, 70, 80, 90, and 100%) at 30 min intervals. IBs were then moved into a vacuum dryer for 24 h to remove ethanol. Samples were mounted on an appropriate stub and sputter-coated with gold in a vacuum gold sprayer. The coated samples were observed at magnifications of 350 and 4500 times while maintaining an acceleration voltage of 10 kV.

### 2.5. Sensory Evaluation

Sensory evaluation was performed by nine panelists who were experienced in sensory analysis of silver carp fillets and IBs. According to the method of Carmack et al. [21], a 9-point scale was used to evaluate of hardness and sharpness of softened IBs (9 = extremely soft to 1 = extremely hard/sharp) and acidic flavor and texture of fillets (9 = unacceptable to 1 = extremely acceptable).

### 2.6. Determination of Hardness

The hardness of IBs was measured using a CT3 texture analyzer (Brookfield, Middleboro, MA, USA). IBs with a morphology that was one-end-unequal-biforked (Y) were selected for hardness determination. IBs were trimmed to 13 mm and fixed with a self-designed fixator that forced IBs to be vertical (Supplementary Figure S1). Each trimmed sample was compressed twice to 30% of the sample’s original height at 1 mm/s speed and a 2 g trigger force using a TA40 cylindrical-shaped probe. Hardness was calculated using Texture Loader software version 1.0 (Brookfield, Middleboro, Middleboro, MA, USA).

### 2.7. Determination of Calcium, Phosphorus, and Collagen Content

Calcium and phosphorus contents of IBs were determined by inductively coupled plasma-mass spectrometry (ICP-MS, Agilent ICPOES730, Agilent Technologies, Santa Clara, CA, USA). A 0.1 g sample was added to 5 mL 69% nitric acid (*v*/*v*) followed by heating to 150 °C until all IBs were dissolved totally. The solution was diluted to 1 L before being evaluated by ICP-MS. The method was carried out under working conditions with an RF power of 1000 W and the following plasma condition: 15 L/min plasma, 1.5 L/min auxiliary gas, and 0.75 L/min nebulizer gas in an axial mode using linear calibration.

Collagen content of IBs was determined by measuring hydroxyproline content. A 0.1 g IBs sample was hydrolyzed with 6 M HCl (1 mL) at 110 °C for 5 h. Hydroxyproline content in the hydrolysate was analyzed according to the instructions in the hydroxyproline assay kit.

### 2.8. Fourier Transform Infrared Spectroscopy (FTIR)

The spectra of IBs were recorded using the Nicolet IS10 FTIR spectrometer (Thermo Fisher Scientific Inc., Waltham, MA, USA), according to the method of Wu et al. [22] with minor modifications. IBs and KBr (Merck, Giessen, Germany) were dried in a drying oven at 50 °C overnight prior to analysis. IBs were then grounded to powder in liquid nitrogen. One milligram of IBs powder was mixed with 100 mg of dry KBr, grounded manually using an agate mortar and pestle, and then compressed uniaxially into pellets. The FTIR spectra were collected after background and baseline correction using the transmittance mode with a scanning range of 4000–400 cm^−1^ wavenumber, at a resolution of 4 cm^−1^. The peak signals in the spectra were analyzed using Omnic software (Thermo Electric Corporation, Chicago, IL, USA).

### 2.9. Statistical Analysis

All measurements were performed in triplicate. All data were expressed as means ± standard deviation (SD). Significant differences among samples were analyzed by SPSS 18.0 software (Chicago, IL, USA) based on one-way analysis of variance (ANOVA) using Duncan’s multiple range test, and the means were considered significantly different at *p* < 0.05. The normality and homoscedasticity were tested with Shapiro–Wilk and Levene’s tests. The length of holes on the surface of IBs shown in SEM was measured by imageJ software. Origin 9.0 software (Origin-Lab, Northampton, MA, USA) was used for data plotting.

## 3. Results and Discussion

### 3.1. Number and Morphology of Silver Carp IBs

**Figure 1:**
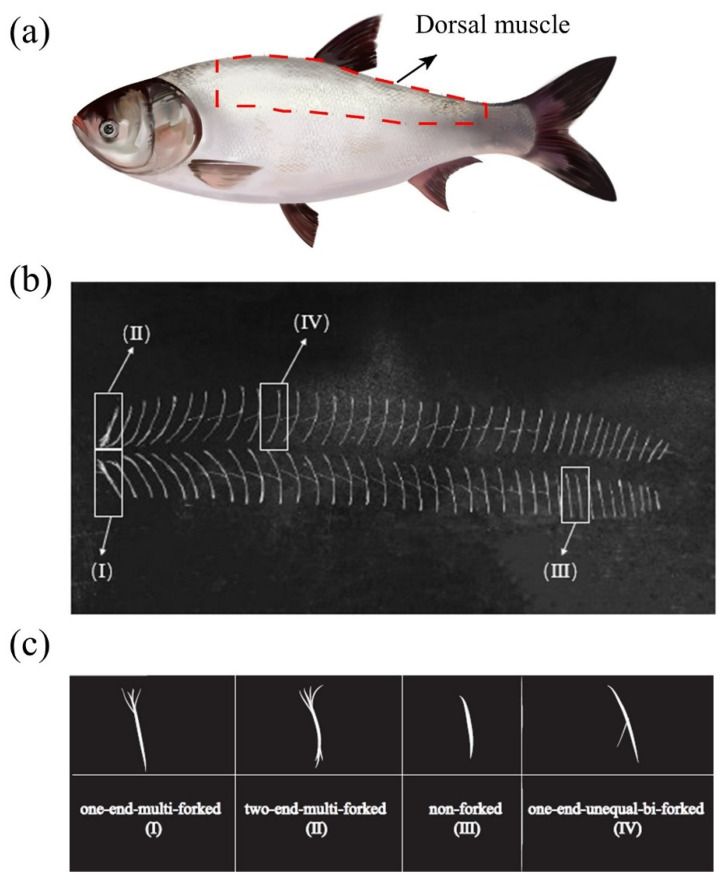
(**a**) The site of the IBs collected on silver carp, (**b**) all intermuscular bones (IBs) from dorsal muscle, and (**c**) the morphologic patterns of IBs of silver carp.

There are three main types of IBs in silver carp muscle: epineurals, epicentrals, and epipleurals, which are in different physical locations in the fish and ossified differently [23]. For consistency, all IBs that we studied in the present study were epineurals from silver carp dorsal muscle (Figure 1a). The total number of epineurals in dorsal muscle was 76 (Figure 1b), and the epineurals included four types (Figure 1c): one-end-multi-forked (Ⅰ), two-end-multi-forked (Ⅱ), non-forked (Ⅲ), and one-end-unequal-bi-forked (Ⅳ). The number of type Ⅰ, type Ⅱ, type Ⅲ, and type Ⅳ IBs was 5, 12, 18, and 41, respectively, and these numbers were consistent with Li et al. [24]. Because the largest proportion (53.94%) of IBs was type Ⅳ, we selected type Ⅳ for further analysis.

### 3.2. Sensory Evaluation

The softening effect of acetic acid on IBs was determined using sensory evaluation; the results are shown in Table 1. For IBs (both in vitro and in vivo), significantly (*p* < 0.05) higher scores were observed when treated with higher concentrations of acetic acid, which indicated that acetic acid softened IBs in a dose-dependent way. Moreover, the results indicated that acetic acid in high concentrations had a negative effect on the flavor and texture of fillets. The sensory scores of fillets treated with 0.5 mmol/L acetic acid were significant (*p* < 0.05) lower than those treated with 0.25 or 0 mmol/L, and the scores of 1 mmol/L treated fillets were significant (*p* < 0.05) lower than those treated with 0.5 mmol/L. This could be explained by that acetic acid weakened connective tissue and denatured myofibrillar protein, which changed the texture of fillets, and acetic acid in higher concentrations induced a much less acceptable acidic taste of fillets. IBs within fillets were softened completely by 1 mmol/L acetic acid; however, the connective tissue and myofibrils of the fillets collapsed, which affected the texture even more (Table 1). The flavor of 1 mmol/L treated fillets had an undesirable sour taste compared with non-treated fillets. Fillets that were treated with 0.25 mmol/L acetic acid had an acceptable flavor and texture but the IBs within them were not well-softened. The optimal concentration of acetic acid for softening IBs in vivo was 0.5 mmol/L. Fillets that were immersed in 0.5 mmol/L acetic acid had an acceptable flavor, firm texture, and well-softened IBs. Acetic acid had a dose-dependent effect on softening IBs, and 0.5 mmol/L acetic acid had a great potential to be used to produce silver carp fillets with softened IBs in the aquatic processing industry.

### 3.3. Effect of Acetic Acid Concentration on the Hardness of IBs

**Figure 2:**
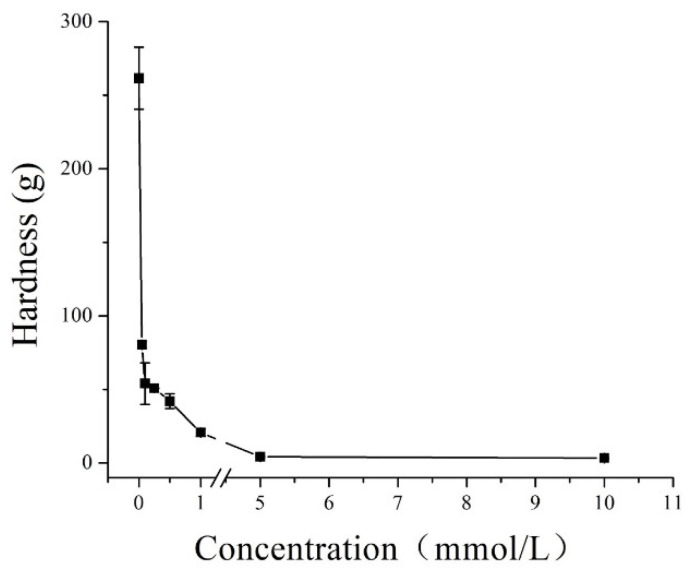
Effect of immersion of acetic acid in different concentrations on the hardness of intermuscular bones (IBs).

To find out the relationship between immersion conditions and the softening of IBs, acetic acid concentrations for softening IBs (in vitro) was varied from 0 to 10 mmol/L. Those ranges were chosen to avoid adding an extremely acidic flavor to fillets. The hardness of IBs decreased significantly from 261.7 to 3.3 g when the acetic acid concentration was increased from 0 to 10 mmol/L for 12 h (Figure 2). The rapid decrease in hardness (261.7 g to 3.8 g) from 0 to 5 mmol/L was most likely caused by the dissolution of collagen and HAP [25]. Meanwhile, the rapid decrease in hardness occurred even at a low concentration (0.05 mmol/L) of acetic acid, but the slow decrease occurred at higher concentrations (5–10 mmol/L), and the hardness remained lower than 4.0 g. Because all concentrations used in our study were relatively low, it may have been that only a small part of acid-soluble collagen and HAP in IBs could be dissolved. However, the exact softening mechanism was still unclear and is investigated in the following sections of this paper, which include SEM, minerals content, and FTIR analyses.

### 3.4. Changes in IB Microstructure

**Figure 3:**
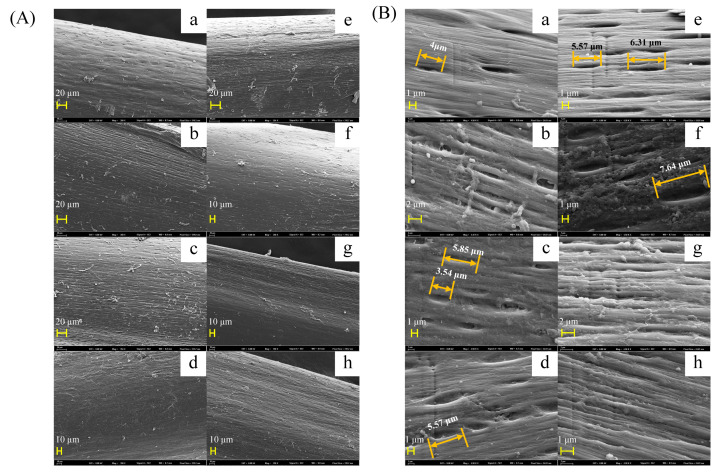
SEM micrographs of intermuscular bones after being immersed in different concentrations of acetic acid: (**A**) under 350× magnification; (**B**) under 4500× magnification. Corresponding acetic acid concentrations are: a. 0 mmol/L, b. 0.05 mmol/L, c. 0.1 mmol/L, d. 0.25 mmol/L, e. 0.5 mmol/L, f. 1 mmol/L, g. 5 mmol/L, h. 10 mmol/L.

SEM was used to determine the microstructural changes in IBs induced by acetic acid. The control IB sample (Figure 3(Aa)) exhibited a smoother surface with smaller holes compared with acetic acid-treated samples (Figure 3). IBs are composed of fibrous bone layers, each of which is composed of organic and inorganic substances, primarily collagen and HAP [26]. In each fibrous layer, collagen molecules are parallel to each other, and holes are formed due to their staggered arrangement [27]. HAP has a crystalline structure and is located in the holes, and the crystals grow in length along the collagen long axis and in width along the channels until they emerge from the holes and cover the collagens [7,8,9]. Holes on the surface of control IBs were oval-shaped, the lengths of the long axes and short axes of holes were 2.48–4.52 μm and 0.64–0.88 μm, respectively (Figure 3(Aa)). The holes enlarged with an increase in acetic acid concentration, but the width remained almost unchanged. The long axes of holes on the surface of 0.5 mmol/L acetic acid-treated IBs were 5.68–8.78 μm, twice as long as the holes in the control group (Figure 3(Ae)). The shape of the holes was still oval-shaped until the acetic acid concentration was ≥0.5 mmol/L (Figure 3B). Acetic acid ≥ 1 mmol/L caused the exposure of fibrous bone layers, which confirmed the fact that acetic acid dissolved HAP and collagen from IBs. Moreover, the results (Figure 3) also revealed that the elution of HAP and collagen caused by acetic acid was dose-dependent, which was consistent with the decreased hardness depicted in Figure 2.

### 3.5. Calcium, Phosphorus, and Collagen Contents

**Figure 4:**
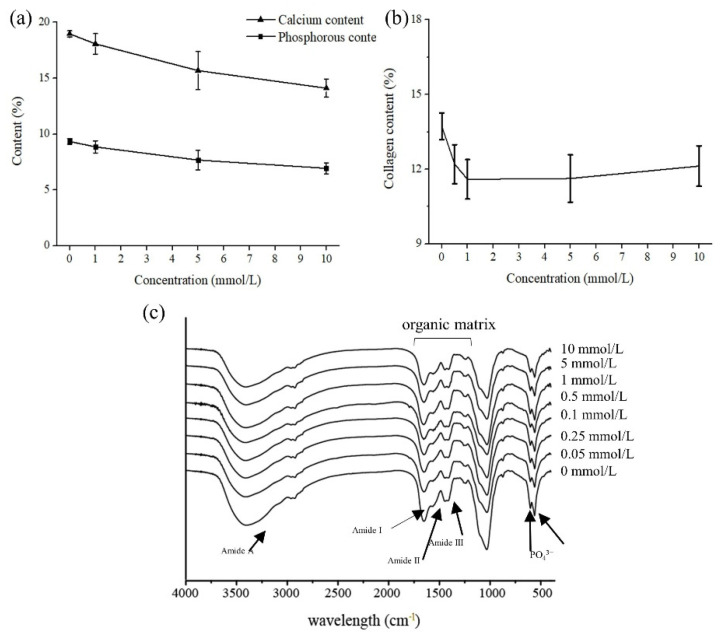
Changes in (**a**) calcium and phosphorous contents, (**b**) collagen content, and (**c**) FTIR spectra of intermuscular bones of silver carp after immersion by acetic acid in different concentrations.

Calcium and phosphorus are the major minerals in HAP (Ca_10_(PO_4_)_3_(OH)_2_). The determination of the calcium and phosphorus contents of acetic acid-treated IBs could provide more direct evidence for the dissolution of HAP (Figure 4a). The initial contents of calcium and phosphorus in IBs were 18.97% and 9.34%, respectively (Figure 4a). The ratio of calcium to phosphorous was almost 2:1, which indicated the presence of B-type carbonate hydroxyapatite in silver carp IBs. Because the molar ratio of calcium and phosphorus was 1.67, as the stoichiometric formula is Ca_10_(PO_4_)_3_(OH)_2_, the higher molar Ca/P ratio obtained in this study can be attributed to the presence of carbonate ions that substituted for phosphate [28]. As the acetic acid concentration increased, the content of both mineral contents decreased continuously, but the ratio was almost unchanged. The results suggested that the dissolution of HAP occurred in both the form of phosphates and carbonates.

Collagen content changes induced by different concentrations of acetic acid are shown in Figure 4b. The content of collagen was determined by measuring the content of hydroxyproline. Collagen content decreased significantly (*p* < 0.05) (from 13.72% to 11.59%) as the acetic acid concentrations increased from 0 to 1 mmol/L. However, the collagen content did not decrease (*p* > 0.05) when higher concentrations (5 and 10 mmol/L) of acetic acid were used. The results greatly coincided with the decrease in hardness, as shown in Figure 2, indicating that diluted acetic acid only dissolved a small part of collagen from IBs, and the hardness that decreased at low concentrations of acetic acid was predominantly associated with the dissolution of HAP. More collagen can be extracted by higher concentrations of acetic acid. For example, Nagai and Suzuki [29] extracted collagen from the bones of five species of fish with 0.5 mol/L acetic acid in a very high yield (skipjack tuna, 42.3%; Japanese sea bass, 40.7%; ayu, 53.6%; yellow sea bream, 40.1%; and horse mackerel, 43.5%, on the basis of lyophilized dry weight).

### 3.6. FTIR

FTIR is a powerful tool for establishing the important material properties that contribute to bone strength [30]. The FTIR spectra of the acetic acid-treated IBs were shown in Figure 4c. All FTIR spectra showed absorption bands that were characteristic of an organic matrix and minerals. The organic matrix was observed as spectral peaks within the amide range from 1750 to 1250 cm^−1^ [31]. Amide that exhibited bands with peaks at 1650–1635 cm^−1^, 1550–1535 cm^−1^, and 1240 cm^−1^ were regarded as Amide I, Amide II, and Amide III, respectively [30]. As shown in Figure 4c, the absorption ratio between and 1240 cm^−1^ (Amide III) and 1454 cm^−1^ is approximately equal to 1.0, suggesting the absorption of the triple helical structure of collagen [32]. A slight decrease in the ratio, as shown in Figure 4c, indicated acetic acid caused collagen loss from IBs. Peaks assigned to the stretching vibration, symmetric bending vibration, and nonsymmetric bending vibration of PO_4_^3–^ were 1033, 605.54, and 565 cm^−1^, respectively. With the increase in acetic acid concentration, the absorption of these three bands decreased continuously, which indicated a dramatic decrease in HAP content. Acetic acid caused the rearrangement of intermolecular bonds of collagen [33]. The range of 3400–3440 cm^−1^ is known as the amide A band and is associated with N-H stretching [32]. A slight left-shift of the amide A band in Figure 4c suggested the loss of hydroxyl groups in collagen; when the N-H group of a protein was involved in hydrogen bonding, the amide A band shifted to lower frequencies [34]. In bones, collagen and HAP strongly bind to each other by sharing hydroxyl groups, which are abundant in collagen [35]. The crosslinks (formed by hydroxyl groups) of collagens, and between collagens and HAP, are essential for bones to possess a sufficient deflection capacity, strength, and stiffness [36].

**Figure 5:**
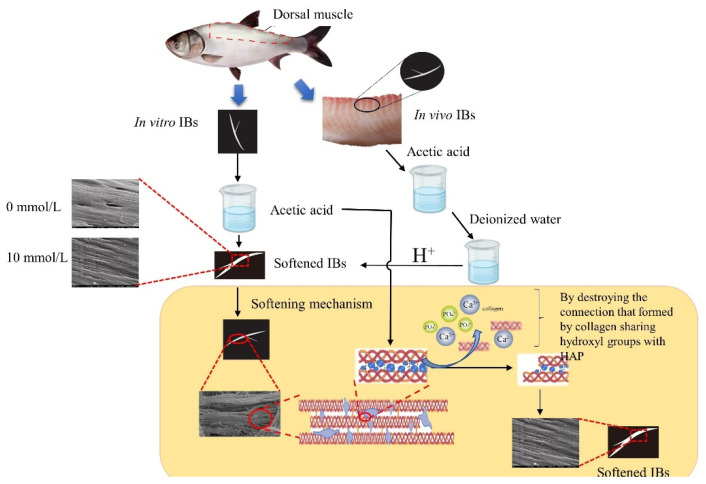
Proposed mechanism of acetic acid-induced breakage of hydroxyl groups of collagens, leading to softening of IBs.

Based on the results of SEM, minerals and collagen contents determination, and FTIR spectra, we proposed a mechanism of diluted acetic acid softening IBs from silver carp (Figure 5). Acetic acid undermined the hydroxyl groups that connected the collagens, as well as collagen and HAP, leading to the release of partial collagens and HAP. The release further causes a hardness decrease in IBs. Moreover, mineral and collagen contents determination suggested that this hardness decrease was predominantly associated with the dissolution of HAP since the concentrations of acetic acid used in the present study were relatively low (0–10 mmol/L).

## 4. Conclusions

This study clarified the softening mechanism of IBs treated with acetic acid (in vitro). The process was characterized by acetic acid that weakened connections formed by hydroxyl groups among collagens and between collagen and HAP, which further caused the dissolution of partial collagen and HAP from IBs, in turn causing the softening of IBs. The dissolution of HAP contributed more to IBs softening than collagen dissolution at a diluted acetic acid concentration. Moreover, the flavor and texture of silver carp fillets were almost unchanged, but the IBs in the fillets were well softened after being treated with 0.5 mmol/L acetic acid. Therefore, the present study provided a strategy for producing silver carp fillets with softened IBs, and the fillets have the potential to serve as raw materials for further processing. Future investigations will focus on the effect of acetic acid on other characteristics of fillets, such as antibacterial characterization, protein denaturation, and additional sensory characteristics after being heated, which will provide a comprehensive understanding of changes in acetic acid-treated fillets for the fishery industry.

## Figures and Tables

**Table 1:** Sensory attributes of IBs and fillets as immersed by acetic acid in different concentrations.

In Vitro					In Vivo				
Concentration (mmol/L)	Hardness	Sharpness	Total Scores	Concentration (mmol/L)	Flavor of Fillets	Texture of Fillets	Hardness of IBs		
							Hardness	Sharpness	Total Scores
10	8.61 ± 0.70 a	8.78 ± 0.44 a	17.39 ± 1.11 a	1	3.30 ± 1.24 a	1.60 ± 0.50 a	8.14 ± 1.21 a	8.43 ± 0.79 a	16.57 ± 1.99 a
1	5.56 ± 1.33 b	6.56 ± 1.33 b	12.11 ± 2.37 b	0.75	5.48 ± 1.32 b	3.08 ± 0.87 b	7.90 ± 1.45 a	8.30 ± 1.16 a	16.20 ± 2.53 b
0.5	6.00 ± 1.12 b	6.44 ± 1.33 b	12.44 ± 2.13 b	0.5	6.05 ± 0.87 b	6.25 ± 0.82 c	6.18 ± 1.40 a	6.64 ± 1.29 a	13.30 ± 1.89 c
0.25	5.33 ± 1.94 b	5.89 ± 1.27 b	11.22 ± 3.03 b	0.25	7.03 ± 0.67 c	6.83 ± 1.13 c	3.56 ± 1.13 b	3.00 ± 1.32 b	6.56 ± 2.40 d
0.05	3.44 ± 1.01 c	4.78 ± 1.30 b	8.22 ± 2.05 c	0	7.20 ± 0.71 c	7.60 ± 0.88 d	2.80 ± 1.14 b	2.80 ± 1.14 b	5.50 ± 2.12 d

Mean values ± SD are calculated based on three replications. Different lowercase superscript letters in the same row indicate a statistical difference at *p* < 0.05.

## Data Availability

Data are contained within the article and the Supplementary Materials.

## Supplementary Materials

The following are available online at https://www.mdpi.com/article/10.3390/foods11010001/s1, Figure S1: Schematic of the fixation of intermuscular bones (IBs) for detecting hardness of silver carp IBs.
