# Supplementary material for: Diluted Acetic Acid Softened Intermuscular Bones from Silver Carp (Hypophthalmichthys molitrix) by Dissolving Hydroxyapatite and Collagen
Source: Foods. 2021 Dec 21;11(1):1. doi: 10.3390/foods11010001 (PMC8749972; doi:10.3390/foods11010001)
Supplement: Supplementary file 1 [file foods-11-00001-s001.zip › foods-1498177-supplementary.pdf]

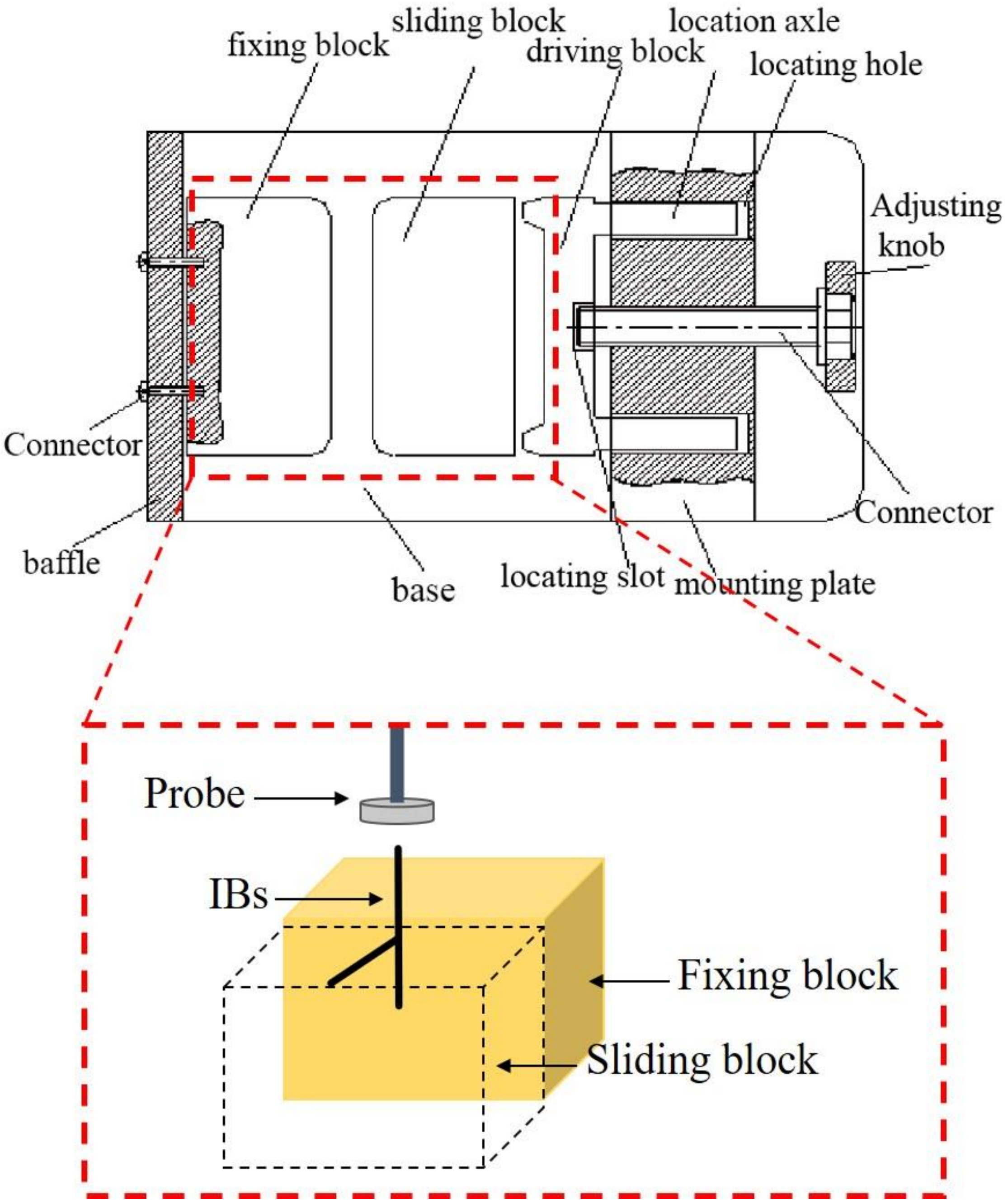

Figure S1. Schematic of the fixation of intermuscular bones (IBs) for detecting hardness of silver carp IBs.
